# Spaceflight Modulates the Expression of Key Oxidative Stress and Cell Cycle Related Genes in Heart

**DOI:** 10.3390/ijms22169088

**Published:** 2021-08-23

**Authors:** Akhilesh Kumar, Candice G. T. Tahimic, Eduardo A. C. Almeida, Ruth K. Globus

**Affiliations:** 1 Space Biosciences Division, NASA Ames Research Center, Mail Stop 288-2, Moffett Field, CA 94035, USA; akhilesh482@gmail.com (A.K.); e.almeida@nasa.gov (E.A.C.A.); 2 Department of Biology, University of North Florida, Jacksonville, FL 32224, USA; c.tahimic@unf.edu

**Keywords:** spaceflight, microgravity, heart, gene expression, oxidative stress, *Nfe2l2*, NADPH oxidase, *Ptgs2*, *Cdkn1a*, *Myc*

## Abstract

Spaceflight causes cardiovascular changes due to microgravity-induced redistribution of body fluids and musculoskeletal unloading. Cardiac deconditioning and atrophy on Earth are associated with altered *Trp53* and oxidative stress-related pathways, but the effects of spaceflight on cardiac changes at the molecular level are less understood. We tested the hypothesis that spaceflight alters the expression of key genes related to stress response pathways, which may contribute to cardiovascular deconditioning during extended spaceflight. Mice were exposed to spaceflight for 15 days or maintained on Earth (ground control). Ventricle tissue was harvested starting ~3 h post-landing. We measured expression of select genes implicated in oxidative stress pathways and *Trp53* signaling by quantitative PCR. Cardiac expression levels of 37 of 168 genes tested were altered after spaceflight. Spaceflight downregulated transcription factor, *Nfe2l2 (Nrf2)*, upregulated *Nox1* and downregulated *Ptgs2*, suggesting a persistent increase in oxidative stress-related target genes. Spaceflight also substantially upregulated *Cdkn1a* (*p21*) and cell cycle/apoptosis-related gene *Myc*, and downregulated the inflammatory response gene *Tnf*. There were no changes in apoptosis-related genes such as *Trp53*. Spaceflight altered the expression of genes regulating redox balance, cell cycle and senescence in cardiac tissue of mice. Thus, spaceflight may contribute to cardiac dysfunction due to oxidative stress.

## 1. Introduction

Responses to spaceflight include cardiovascular deficits, loss of bone and muscle mass, compromised immune function, chromosomal aberrations and metabolic changes [1,2,3]. With future lunar and interplanetary space missions, astronauts will stay in space for longer periods, presenting increased cardiovascular risk to the crew. Therefore, a better understanding of molecular mechanisms that mediate physiological responses to the space environment is needed.

Entering into microgravity causes a cephalad shift of body fluids which leads to increased heart stroke volume, a rise in left ventricular end diastolic dimensions and a fluid shift between the vasculature and interstitium [4,5,6]. The fluid shift invokes compensatory endocrine and renal mechanisms [6]. These alterations can diminish the capability of the heart to cope with gravitational stress immediately after returning to Earth, leading to orthostatic intolerance, as previously documented in some astronauts [7].

Gravitational unloading in space also contributes to cardiovascular deconditioning [8], despite vigorous on-orbit exercise programs implemented on the International Space Station (ISS). Both spaceflight and head-down-tilt bed rest, a ground-based model for human spaceflight, can cause reduced plasma volume and left ventricular atrophy [9,10]. A reduction in myocardial interstitial fluid may contribute to lower myocardial mass following microgravity exposure [11]. In rodents, 14 days of spaceflight was reported to reduce cardiac myocyte size relative to ground controls, which is consistent with cardiac atrophy [12]. In contrast, spaceflight for one week did not affect cardiac mass [13], although it is possible that this period is too short to cause demonstrable cardiac atrophy. Hindlimb unloading (HU), a ground-based rodent model for simulating weightlessness, is reported to cause cardiac atrophy in some studies [14,15], but not in others [13], thus further work in this area is needed.

In addition to gravitational unloading, long term habitation in space will expose astronauts to low doses of ionizing radiation, which may contribute to both cardiac and vascular changes in oxidative pathways leading to tissue dysfunction [16,17,18]. In fact, spaceflight can cause substantial changes in expression of genes and biomarkers related to metabolic and oxidative stress pathways in various tissues of both astronauts and animals [19,20,21,22,23,24,25,26,27]. The relative contributions of the various complex factors of habitation in space, including microgravity and ionizing radiation, to observed changes in oxidative stress-related pathways are not yet understood.

Activation of cell cycle arrest and apoptosis signal transduction pathways are major stress responses important for defending against many physiological and environmental challenges, including tissue oxidative stress. Spaceflight can alter expression of various cell cycle arrest and apoptosis regulatory genes [28,29] including CDKN1A (also known as P21), a cyclin dependent kinase inhibitor that mediates cell cycle arrest via cyclin interactions and apoptosis via TRP53. In addition, CDKN1A/P21 is a negative regulator of tissue regeneration in mammals, and is known to interact with NFE2L2 (aka NRF2), a master transcriptional regulator of oxidative defense genes. Spaceflight elevates *Cdkn1a* expression in bone tissue [30], liver and kidney [31] as well as cultured cells [29], implicating activation of CDKN1A as a pathway invoked in multiple tissues in response to the space environment.

We hypothesized that spaceflight leads to activation of oxidative stress and cell cycle/apoptosis-related pathways in the heart. To begin to test this hypothesis, we used thematic PCR arrays to identify the changes in expression levels of genes related to oxidative stress and cell cycle/apoptosis (*Trp53*) signal transduction pathways in mice exposed to 15 days of spaceflight on the STS-131 space shuttle mission. Our gene expression findings in heart are novel and consistent with activation of several molecular pathways, including those related to oxidative stress, cell cycle regulation, inflammation and DNA damage repair, which ultimately may contribute to cardiac dysfunction during long duration spaceflight.

## 2. Results

### 2.1. Body Mass of Animals Pre- and Post-Flight

**Figure 1:**
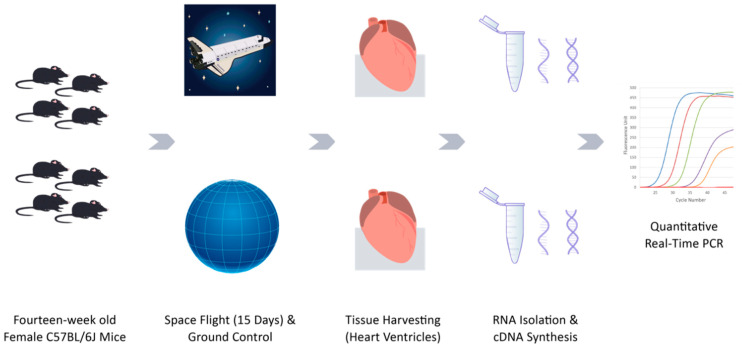
Schematic diagram of experiment design and workflow.

Immediately after landing, all flight (FLT) and ground (GRD) control mice were determined to be in good condition by veterinary examination. Figure 1 shows the schematic of the experiment design and workflow. The average weight loss of FLT mice over the duration of the experiment was 2.0 g (−9.1%, SD = 2.1 g, *p* = 0.013) whereas GRD control mice lost an average of 0.7 g (−3.4%, SD = 0.9 g, *p* = 0.096); the difference in body weight loss averages for FLT versus GRD was not significant (*p* = 0.143). FLT habitats showed an average of 10% less food remaining, and 24% less water remaining than GRD habitats. However, measured values for food and water utilization after flight may not precisely reflect actual consumption by mice due to possible differences in food breakdown and water spillage in FLT versus GRD habitats.

### 2.2. Spaceflight Altered Expression of Oxidative Stress Related Genes

**Figure 2:**
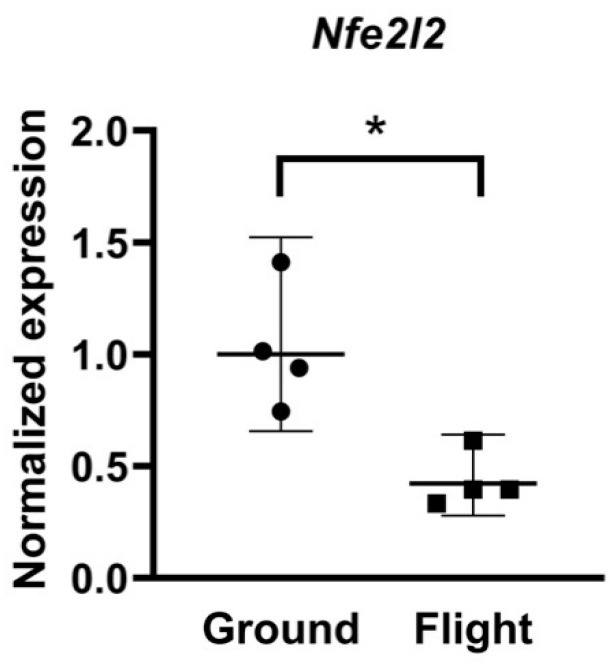
Normalized expression levels of *Nfe2l2* as measured by a separate RT-PCR assay. Each data point is shown. The middle lines indicate the geometric mean (normalized expression of 1 for Ground Control and 0.42 for Spaceflight group). Outer lines show the 95% confidence intervals. * Statistically significant by Mann–Whitney U test at *p* < 0.05, n = 4. The log2 (linear) normalized expression from the Ground Control and Flight groups were used in the Mann–Whitney U test.

Comparable quantities (average of 38.5 μg RNA per tissue sample) and high qualities of total RNA were obtained from all the FLT and GRD samples. To determine if spaceflight altered the expression of genes regulating redox balance in cardiac tissue, oxidative stress PCR array was performed on 84 genes related to oxidative metabolism (Supplementary Table S1), and this data set was supplemented with real time PCR analysis of *Nfe2l2* expression levels (Figure 2). Gene expression levels of FLT and GRD controls showed a trend towards upregulation of a majority of those genes which were altered by spaceflight (Table 1). Out of the 84 genes on the array, 11 genes were upregulated and 3 genes were downregulated in FLT samples relative to GRD (*p* < 0.05) (Table 1).

Specifically, there was a marked 6.6-fold upregulation in expression of NADPH oxidase 1 (*Nox1*), suggesting an increase in production of superoxide and/or hydrogen peroxide. Additionally, expression of Prostaglandin endoperoxidase synthase 2 (*Ptgs2*), which acts as peroxidase, was downregulated by 3.4-fold. The upregulation of *Nox1* and downregulation of *Ptgs2* may reflect elevated oxidative stress within heart tissue of FLT mice relative to GRD controls. In addition, *Txnip* was upregulated by 1.9-fold and *Fancc* was downregulated 1.6-fold in FLT samples. *Ncf2*, which codes for a component of the NADPH oxidase complex that generates superoxide, was moderately downregulated by spaceflight (−1.5-fold). Other antioxidant genes (*Sod1*, *Cat*, *Prdx6*, *Gsr*, *Tpo*, *Txnrd3*, *Slc41a3*) and oxidative stress-responsive genes (*Xpa*, *Prnp*) were moderately affected by spaceflight (1.2-2.4 fold, *p* < 0.05) (Table 1). There were no changes in the expression levels of 12 genes related to oxygen transport (Supplementary Table S1). In contrast, there was a 2.4-fold reduction in expression of the global transcription factor, *Nfe2l2*, in FLT compared to GRD animals (Figure 2). Thus, the expression of redox-related genes was differentially regulated as a consequence of exposure to the spaceflight environment.

### 2.3. Spaceflight Altered Expression of Cell Cycle/Proliferation Related Genes

The expression levels of 84 genes involved cell cycle and apoptosis signal transduction were analyzed by *p53* Signaling Pathway PCR array (Supplementary Table S2). Out of 84 genes, 13 genes were upregulated and 10 genes were downregulated in FLT compared to GRD controls (Table 2, *p* < 0.05). Results show that genes related to cell cycle regulation were altered due to spaceflight. Specifically, cell cycle inhibitors such as *Cdkn1a* were upregulated 6.8-fold while *Cdk1* was downregulated 4.1-fold, suggesting diminished growth and proliferation signals in FLT samples compared to GRD. *Myc* was upregulated 3.9-fold (*p* < 0.05). Genes involved in inflammatory responses, including *Tnf*, *Tnfrsf10b* (Tnf receptor superfamily, member 10) and *Traf1*, were downregulated (9.9-, 1.9- and 2.3-fold decrease, respectively) in spaceflight samples. Out of the 14 genes analyzed for DNA damage repair, four genes were modestly upregulated (*Atr*, *Xrcc5*, *Ercc1*, *Apex1*; 1.1–1.3-fold) (Table 2, *p* < 0.05). There were no significant changes in apoptosis-related genes including *Trp53*, *Bax*, *Bcl2*, *Casp2* or *Casp9* (Supplementary Table S2).

### 2.4. Gene Networks and Disease Predictions

**Figure 3:**
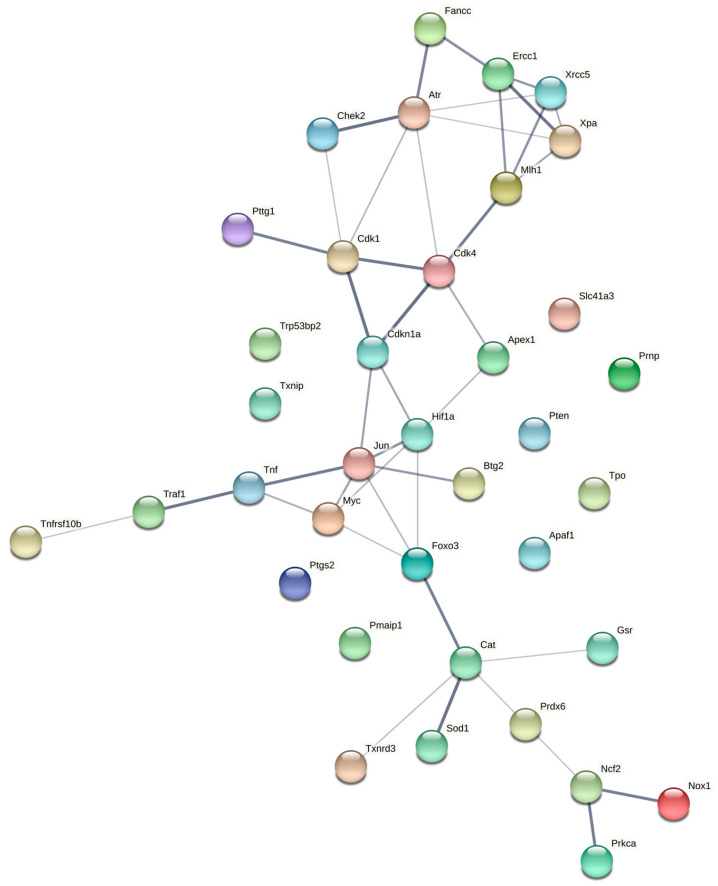
Predicted molecular interactions among DEGs from an oxidative stress PCR Array and *p53* Signaling Pathway PCR Array using String Db (Refer to the Methods section for details on analysis). Thicker lines connecting genes represent greater confidence in molecular interactions.

We made use of a computational approach to gain insight into possible protein–protein interactions among the differentially expressed genes. Analysis using String Db revealed potential interactions between the oxidative stress response and *Trp53* pathways (Figure 3). A number of oxidative stress response DEGs formed a molecular interaction network including *Nox1* and *Sod1* with Catalase serving as a central node. DEGs from the *Trp53* signaling pathway also formed molecular interaction networks involving cell cycle-related genes and transcription factors. For the *Trp53* pathway, *Cdk1, Cdk4, Cdkn1a* and *Atr* served as central nodes in a molecular network that included *Chek2*, *Fancc*, *Ercc1*, *Xrcc5* and *Xpa1* among others. Interestingly, the oxidative stress response and *Trp53* pathways were linked by central nodes *FoxO3*, *Jun*, *Myc*, and *Hif1a*, all of which code for major transcriptional regulators. This finding suggests global transcriptomic changes in the heart response to spaceflight.

We also utilized a computational approach to gain insight into possible disease phenotypes associated with spaceflight-induced changes in gene expression. Functional enrichment analysis of oxidative stress array DEGs using Toppfun revealed enrichment of genes linked to hypertensive disease, atherosclerosis, cerebrovascular accident and decompensation (Supplementary Table S3). Using a similar analysis approach, *p53* array DEGs revealed potential links to a number of cancers including sarcoma, immune suppression, impaired glucose tolerance and cardiovascular pathologies including hypertensive disease, acute myocardial infarction, and atherosclerosis (Supplementary Table S4).

## 3. Discussion

Our findings provide new insight into how the heart responds at the molecular level to the novel environment of space. Spaceflight experiments using rodents present unique practical challenges though similar benefits to rodent studies on Earth. The animals flown in space during this particular mission were deemed healthy, as assessed by veterinary examination after return to Earth, and the finding that animal body weights were comparable in FLT mice compared to GRD controls at the termination of the study. Further, numerous scientific reports from these mice describe related results obtained studying other organ systems (e.g., [30,32,33]). The RNA samples recovered from the hearts were consistent and of high quality. Therefore, we conclude that the results we obtained from this mission were valid. 

Whereas cardiovascular physiology has been studied intensively in astronauts, analysis of cardiac changes at the tissue level has been more limited [12,13,34,35,36]. Goldstein et al. [12] report that spaceflight (14 days) reduces the cross-sectional area of papillary cardiomyocytes in rats relative to ground controls, with ultrastructural changes consistent with cardiac atrophy. Short duration (6 days) spaceflight upregulates activity and mRNA expression of the mitochondrial enzyme, malate dehydrogenase in rat hearts, and displays a pattern of mitochondrial gene expression changes discrete from those in skeletal muscle [34]. More recently, long duration spaceflight (37 days) was shown to alter expression of methylation-related genes in the heart of adult mice, but not cytoskeletal protein levels. Interestingly, lifetime exposure of *Drosophila* to microgravity leads to diminished cardiac size, cardiac dysfunction, altered remodeling, and proteosomal abnormalities [36]. Furthermore, spaceflight causes cardiac progenitor cells in culture to display a gene expression profile consistent with early, stem-like function along with increased oxidative stress and re-entry into the cell cycle [37]. Thus, our study on expression levels of oxidative stress and cell cycle-related genes provides novel findings on how the heart responds to spaceflight at the tissue level.

### 3.1. Oxidative Stress-Related Gene Expression in Spaceflight Heart

RT-PCR array data show that expression levels of 14 genes were significantly (*p* < 0.05) altered (11 upregulated and 3 downregulated) in FLT samples as compared to GRD controls. In addition, RT-PCR analysis for *Nfe2l2* revealed lower levels in FLT mice than GRD. *Nfe2l2* is a master transcriptional regulator for oxidative stress response genes, and is rapidly transcribed within seconds following changes in the gravity vector [23]. Other tissues also display reduced *Nfe2l2* expression levels in similar spaceflight experiments entailing tissue recovery after landing [38]. Suzuki et al. recently demonstrated that long duration spaceflight activates *Nfe2l2*-dependent oxidative stress pathways in several tissues of growing mice [39]. Furthermore, *Nfe2l2*-deficient mice are protected from spaceflight-induced oxidative stress-related genes and changes in body weight [39], and also display accelerated transition of muscle fiber type without protecting from atrophy [40].

We also found that spaceflight upregulated *Nox1* 6.6-fold, the change of greatest magnitude in oxidative stress-related genes observed in our study. NOX1 belongs to the NADPH oxidase family of enzymes and functions in generating ROS in a highly regulated fashion [41,42]. NOX1 utilizes NADPH as an electron donor and catalyzes transfer of electrons to molecular oxygen to generate superoxide radicals. Superoxide radicals are short lived and are readily converted into hydrogen peroxide. Thus, increased expression of *Nox1* can cause an increase in the formation of hydrogen peroxide and other reactive oxygen species (ROS) [41,42]. NADPH oxidases are major sources of ROS in the vascular system and are involved in the pathophysiology of many cardiovascular diseases such as hypertension and myocardial infarction [43]. Several recent studies using ground-based models to simulate weightlessness show that hindlimb unloading causes cardiac atrophy, reduces cardiomyocyte size and cardiac dysfunction, as assessed by fractional shortening [14]. Inhibition of NOX1 by apocynin (a NADPH oxidase inhibitor) reduces oxidative stress markers in the heart, and mitigates atrophy and dysfunction in hindlimb unloaded mice, suggesting that *Nox1* plays an important role in mediating cardiac responses to simulated weightlessness [44]. Thus, our results support the hypothesis that increased expression of *Nox1* during spaceflight contributes to oxidative stress and myocardial abnormalities.

Oxidative stress PCR array results showed lower expression of *Ptgs2* by 3.4-fold in FLT samples compared to GRD. PTGS2, also known as Cycloxygenase 2 (COX2), is the key, inducible enzyme in prostaglandin biosynthesis, and acts as both a dioxygenase and as a peroxidase, converting hydrogen peroxide into water. Thus, lower *Ptgs2* expression can lead to inefficient removal of hydrogen peroxide, accumulation of ROS and oxidative damage. PTGS2 is important for the maintenance of healthy cardiac tissue. Conditional cardiomyocyte-specific deletion of the *Ptgs2* gene in adult mice reduces cardiac output, decreases exercise tolerance and increases susceptibility to induced ventricular arrhythmias [45]. Furthermore, human and animal models of myocardial diseases show that *Ptgs2* expression is associated with cardiac tissue damaged by infarction, septicemia and inflammatory heart diseases [46]. In addition to inflammation, the prostaglandin pathway plays an important role in mechanical signaling. In cultured cells activated by serum, spaceflight prevents induction of *Ptgs2* expression, which is avoided by on-orbit centrifugation to replace the gravity vector, implicating sensitivity to a biomechanical mechanism for microgravity-induced activation of the prostaglandin pathway [47]. Inhibition of PTGS2 also impairs recovery of skeletal muscle and bone from hindlimb unloading [48,49]. Thus, regulation of the prostaglandin pathway by spaceflight via changes in *Ptgs2* expression may contribute to both oxidative stress and subsequent recovery on Earth.

*Txnip* was upregulated 1.9-fold and *Fancc* was downregulated 1.6-fold in FLT compared to GRD, which also may contribute to a pro-oxidative milieu in the heart during spaceflight. TXNIP is a negative regulator of thioredoxins (TRXs) [50], which are potent antioxidants, and their downregulation by elevated *Txnip* expression in FLT samples may lead to increased oxidative stress in cardiac tissue. In cultured human endothelial cells (HUVEC), *Txnip* was the most upregulated by spaceflight (33-fold) [51]. *Fancc* codes for an antioxidant and its downregulation can cause sensitivity to oxidative stress-mediated damage to tissue [52].

Our results indicated moderate changes in several antioxidant genes (*Sod1*, *Cat*, *Prdx6*, *Gsr*, *Tpo*, *Txnrd3*, *Slc41a3*) and other oxidative stress-responsive genes (*Xpa*, *Prnp*) (1.2–2.4-fold, *p* < 0.05) (Table 1). The observed elevated expression levels of these antioxidant genes may be due to activation of compensatory mechanisms.

Although the magnitude of differences between FLT and GRD for statistically significant genes were generally modest, together, the findings suggest that spaceflight activates a network of genes responsible for redox signaling and defense against oxidative stress in the heart. 

### 3.2. Cell Cycle and Senescence-Related Gene Expression in Spaceflight Heart

Increased generation of ROS during spaceflight can lead to protein, lipid and DNA damage in tissue and, as is the case with skeletal muscle, may contribute to dysfunction caused by gravitational unloading. ROS are also involved in vital cell signaling pathways such as cell-cycle progression, differentiation, inflammation and apoptosis. Cell cycle progression in eukaryotes is regulated at various checkpoints by the action of specific cyclin dependent kinases (CDKs). The activities of these CDKs are controlled by the positive regulation from cyclins and negative regulation from cyclin dependent kinase inhibitors (CDKIs). Hence, expression levels of genes involved in cell cycle regulation, inflammation, DNA damage repair and apoptosis were further analyzed in hearts by *p53* Signaling PCR array. The *p53* Signaling PCR array includes 24 genes involved in cell cycle regulation. Results indicate that nine of these genes were significantly altered in FLT samples.

We found spaceflight increased *Cdkn1a*/p21 expression 6.8-fold in hearts compared to GRD, consistent with reports for other tissues including liver, kidney, bone and muscle [27,30,31]. *CDKN1A* (also known as P21), is a potent CDK inhibitor and inhibits cell cycle progression at the G1 phase by inhibiting the activity of CDK2 or CDK4. Increases in *p21* levels in bone from mice in this spaceflight experiment spaceflight were previously associated with proliferative arrest in cultured osteoprogenitors [30]. However, the adult mammalian heart itself has limited regenerative ability. Adult *p21* levels in adult heart are thought to contribute to normal cell cycle arrest in differentiated cardiomyocytes, and there is evidence that cardiomyocytes can divide in the adult human heart [53], though it is unlikely that such a small sub-population (1.3% of total) [53] can account for our findings. More likely, the elevation in *p21* caused by spaceflight may contribute to cardiomyopathies [54] via other cellular processes such as DNA repair, autophagy [38,55,56] and senescence. Interestingly, *Myc*, a mitogenic transcription factor gene that promotes cardiac hypertrophy in response to Angiotensin II, was also highly upregulated in the spaceflight heart samples we studied, and its deleterious action can be rescued by *p21* re-expression in *p21*-null mice [57]. Finally, in newborn mammals, *p21* elevation might interfere with cardiomyocyte binucleation, an important heart growth mechanism during post-natal development [58].

PTTG1 (Pituitary tumor-transforming gene 1) regulates the cell cycle by inducing cyclin D3 and repressing *p21* expression [59]. Thus, a decrease in *Pttg1* expression (2.4-fold) observed in FLT samples may contribute to the observed increase in expression of *p21* in FLT samples. In addition to upregulation of cell cycle inhibitors, CDK’s and cyclins such as *Cdk1* and *Cdk4* were downregulated in FLT samples, implicating an overall cell cycle arrest in spaceflight samples.

Our results also indicate some apparently contradictory findings, such as the expression levels of genes for cell cycle arrest proteins, *Chek2* and *Btg2*, were decreased 2.2- and 3.5-fold, respectively in FLT samples, contrary to what is expected for *Chek2* when *Myc* is overexpressed based on what is observed in lymphoma cells [60].

Thus, one of the crucial findings of our study is that spaceflight alters the expression levels of major cell cycle and cell growth regulators. As the samples analyzed consist predominantly (but not solely) of fully differentiated non-dividing cardiomyocytes, this suggests that spaceflight activates *p21*- and *Myc*-related pathways to regulate cell growth, cardiomyocyte function, and potentially cardiomyopathy, although complex interactions such as these remain to be elucidated.

### 3.3. Apoptosis and DNA Damage Repair-Related Gene Expression in Spaceflight Hearts

There were no significant changes in apoptosis-related genes such as *p53*, *Bax*, *Bcl2*, *Casp2* or *Casp9* (Supplementary Table S2). DNA damage repair genes such as *Atr*, *Xrcc5*, *Ercc1* and *Apex1* were only moderately altered in spaceflight (1.1- to 1.3-fold), suggesting widespread DNA damage responses were not occurring at the time of sample recovery. However, we cannot rule out the possibility that spaceflight activates DNA damage and apoptosis pathways in a smaller sub-population of cell types within the heart during spaceflight, as shown previously for exposure to low dose, heavy ion radiation (^16^O, 600 MeV/n) simulating space radiation [61]. Evidence supporting the possibility that spaceflight activates DNA damage pathways and/or apoptosis has been reported for tissues other than heart [62], including the thymus [63,64] and the eye [62,64], and multi-omics analyses reveal increased levels of DNA damage markers in urine and blood from astronauts [26].

### 3.4. Inflammatory Pathway Gene Expression in Spaceflight Heart

Inflammatory response genes, such as *Tnf* (tumor necrosis factor), *Tnfrsf10b* (*Tnf* receptor superfamily, member 10) and *Traf1* (Tnf receptor-associated factor) were significantly downregulated (9.9-, 1.9- and 2.3-fold decrease) in FLT samples compared to GRD controls. *Tnf* is a multifunctional pro-inflammatory cytokine, which regulates a wide variety of biological processes such as inflammation, proliferation, differentiation, apoptosis and cytotoxicity [65]. Space is a hazardous environment and proper immune functioning is crucial for astronaut health and success of space missions. The decreased expression of *Tnf*, *Tnfrsf10b* and *Traf1* observed in our study suggests downregulation of this pro-inflammatory signaling pathway, which may lead to compromised immune function. Our results are consistent with other studies showing spaceflight reduces *Tnf* expression levels in both mice [19,66] and humans [67]. In addition, human T cell cultures on the International Space Station (ISS) show a 15-fold decrement in expression of *Tnf* in T cells and its downstream effectors in microgravity as compared to cells centrifuged at 1g [68]. During a very long duration spaceflight, *TNF*α expression in the blood of astronauts is elevated [24] but after return to Earth (between days 0 and 5) as skeletal muscle enters a recovery phase, *Tnfα* expression rapidly declines along with other cytokines in the *Il6* pathway [69]. Together, these findings implicate a biomechanical, gravity-dependent mechanism for regulating Tnf-related pathways.

Although the radiation exposure of mice on the STS-131 mission is considered too low to account for our results, relatively low doses of particle radiation (<30 cGy) can lead to altered regulation of disease-related pathways and deficits in cardiac function [70,71]. Furthermore, expression of oxidative stress and inflammation markers in the heart caused by low dose gamma radiation exposure is exacerbated when combined with hindlimb unloading [17]. Further study is needed to determine the influence of space radiation in combination with microgravity, on the heart in the spaceflight environment during long duration missions.

### 3.5. Gene Networks and Molecular Signatures of Disease

Computational analysis revealed that changes in a subset of genes that were differentially expressed due to spaceflight were linked to disease, including immune suppression and impairments in glucose homeostasis, consistent with findings in astronauts [72,73]. In addition, a subset of the DEGs resulting from spaceflight were associated with cardiovascular pathologies such as atherosclerosis, cardiovascular decompensation, hypertensive disease and myocardial infarction. Cardiovascular decompensation and volumetric changes in blood flow were consistent with previous observations in crew members during flight [3,8,74]. In a previous study on a subset of ISS crewmembers, mean arterial pressures reportedly do not change significantly during spaceflight. However, cardiac output and stroke volume are increased in flight compared to the seated position on Earth (although comparable to the supine position on Earth) [3]. Thus, our findings were consistent with previous reports on astronauts indicating that spaceflight adversely affects cardiovascular health.

Computational analyses also indicated that spaceflight led to altered regulation of molecular networks within the oxidative damage response and cell proliferation pathways. Our findings suggest that oxidative stress and *Trp35* signaling pathways were linked and that spaceflight alters gene interactions between these two pathways. Furthermore, transcription factors *FoxO3*, *Jun*, *Myc*, and *Hif1a* were predicted to mediate the interaction between these two pathways, making these genes promising targets for mitigating gene network changes in response to spaceflight. *Cat*, *Cdkn1a*, *Cdk1* and *Cdk4* also were predicted to serve as central hubs of gene networks that are altered by spaceflight. Hence, these molecules may be potential targets for the development of countermeasures against spaceflight-induced tissue deficits.

### 3.6. Limitations

For extrapolation of findings from this murine study to humans, there is the inherent limitation of the difference in species as well as a potential influence of the specific strain and sex which were selected as subjects. Factors related to experimental protocol also important to consider when interpreting gene expression findings from this experiment include the fixed duration of the mission (15 days), sample recovery only after animals were returned to Earth (3+ hours after landing), and the fact that ground control animals were housed in flight-matched habitats but were not exposed to similar forces as the flight mice encountered during launch and landing. Therefore, observed changes in gene expression did not solely reflect changes observed during habitation in microgravity, but for rapidly responding genes, such as *Nfe2l2*, also may reflect re-entry and re-adaptation to 1-gravity on Earth, however briefly.

As the scope of our investigation focused on RT-PCR, additional studies are needed to elucidate how spaceflight-induced oxidative stress may mediate cell cycle/growth and function, to gain deeper insight into how the cardiovascular system responds to the space environment and recovers after return to Earth. Furthermore, because the heart consists of multiple cell types, including cardiomyocytes, fibroblasts, endothelial cells, and macrophages, each with a discrete gene expression profile [53], future experiments could focus on spatial transcriptomics to determine the cellular source of specific gene expression changes, as well as the regulation of protein expression together with cardiac function.

## 4. Materials and Methods

### 4.1. Ethics Statement

All animal procedures were conducted in accordance with guidelines described by the National Institute of Health Guide for Care and Use of Laboratory Animals, and approved by all relevant committees, including the Institutional Animal Care and Use Committee (IACUC) at National Aeronautics and Space Administration (NASA) Ames Research Center. The IACUC approval number associated with this animal study was NAS-10-002-Y1.

### 4.2. Animals and Spaceflight

The mice in this study were flown on the Space Shuttle Discovery during the STS-131 mission for the experiment “Mouse Antigen-Specific CD4+ T Cell Priming and Memory Response during Spaceflight”. The heart tissue used in this experiment was an award to E. Almeida as part of the NASA STS-131 Mouse Immunology I Biospecimen Sharing Program (BSP). Female C57BL/6J mice were acclimatized to spaceflight food bars and lixits, but were not placed in Animal Enclosure Module (AEM) habitats until just before spaceflight. Mice were divided into two groups: flight (FLT) and ground (GRD) control. The NASA AEM units are composed of a stainless-steel grid cage, fan blowers, a layered filter system, interior lamps, specialized food bars, and a water box. The AEM units are self-contained habitats that provide group housing with constant ad libitum access to food and water while also providing a waste management system that isolates the animals from their waste. FLT mice were launched at 14 weeks of age then flown in space for 15 days and were not subjected to any experimental procedures during spaceflight.

Synchronous GRD control mice were also housed in AEMs, maintained in an orbital environmental simulator at Kennedy Space Center, and exposed to environmental conditions matched closely to those of FLT mice on the shuttle (temperature, light/dark cycle, humidity, and carbon dioxide levels). FLT and GRD control animals were weighed before flight and immediately after return and were subjected to veterinary examination prior to euthanasia. Food and water consumption of each group were measured upon recovery. Animals were euthanized at 16 weeks of age starting approximately 3 h after landing using isoflurane anesthesia followed by thoracotomy. Euthanasia and dissections proceeded at a pace of one mouse every 15–20 min. The heart was removed and major veins and feed arteries trimmed off. The hearts of four mice per group were bisected into the cephalic and caudal regions, and the caudal halves (ventricles) were frozen in liquid nitrogen for later analysis of gene expression (the remaining hearts from four mice per group were distributed to another BSP investigator). The frozen samples were transported on dry ice to NASA Ames Research Center and stored in liquid nitrogen until analysis.

### 4.3. RNA Isolation and cDNA Synthesis

Total RNA was isolated from frozen heart tissue by homogenization and RNA extraction using TRIzol reagent (Life Technologies, Carlsbad, CA, USA). Tissue samples were homogenized in 1 mL of TRIzol reagent and incubated at room temperature for 5 min. After incubation, 0.2 mL of chloroform was added, the sample agitated vigorously for 30 s and then incubated for 2 min at room temperature. The mixture was then centrifuged at 12,000× *g* for 15 min at 4 °C. Following centrifugation, the upper aqueous phases were separated and incubated with 0.5 mL of isopropanol for 10 min at room temperature. Samples were then centrifuged at 12,000× *g* for 15 min at 4 °C and the RNA pellet was washed with 70% ethanol. The RNA pellet was dissolved in 100 μL of nuclease-free water and further purified using an RNeasy Mini Kit (Qiagen, Valencia, CA, USA), per instructions. The quantities and qualities of purified RNA were determined by measuring absorbance at 260 nm and 280 nm using a NanoDrop 2000 Spectrophotometer (Thermo Scientific, Wilmington, DE, USA). RNA integrity was assessed by running RNA on 0.8% agarose formaldehyde-denaturing gel with ethidium bromide and further visualized by Fluor-S Multimager (Bio-Rad, Hercules, CA, USA).

All RNA samples were analyzed by thematic real time PCR arrays (for Oxidative Stress and *p53*-related pathways) or RT-PCR (for *Nfe2l2*). Although whole transcriptome analysis would be valuable, samples are no longer available for additional analyses.

### 4.4. Quantitative Real-Time PCR Array

An equal amount of RNA (1 μg) was used from FLT and GRD samples to synthesize cDNA using RT^2^ PCR Array First Strand Kit (SABiosciences, Frederick, MD, USA). Real-time PCR was performed using the Oxidative Stress PCR Array (PAMM-065) and *p53* Signaling Pathway PCR Array (PAMM-027) according to the manufacturer’s protocol (SABiosciences, Frederick, MD, USA). Briefly, FLT and GRD cDNA samples were mixed with RT^2^ SYBR Green qPCR Master Mix (SABiosciences, Frederick, MD, USA) and distributed across the 96-well plate array. Each of the 96-well plate arrays contained primers for 84 genes related to oxidative stress or *p53* signaling pathways and control housekeeping genes (HKG). PCR was performed in a thermal cycler (ABI 7500 Standard, Applied Biosystems, Foster City, CA, USA) with initial denaturation at 95 °C for 10 min, followed by 40 cycles of 95 °C for 15 s and 60 °C for one minute. FLT and GRD cDNA samples were also run on control RT^2^ RNA QC PCR Array plates (PAMM-999) to test RNA quality, reverse transcription efficiency, PCR efficiency and genomic DNA contamination.

### 4.5. Nfe2l2 Real-Time PCR

cDNA was synthesized by using QuantiTect Reverse Transcription Kit (Qiagen, Valencia, CA, USA). Briefly, 1 μg of RNA sample was treated with gDNA Wipeout Buffer at 42 °C for 2 min then reverse-transcription was performed using Quantiscript RT Buffer and Quantiscript Reverse Transcriptase as per manufacturer’s instruction. The Real-Time PCR mixture was made using QuantiTect SYBR Green PCR Kit (Qiagen, Valencia, CA, USA). Synthesized cDNA from the previous step was mixed with Quantiscript SYBR Green PCR Mix and *Nfe2l2* primer mixture, per manufacturer’s protocol. Real-time PCR was performed in a thermal cycler (ABI 7500 Standard, Applied Biosystems, Foster City, CA) with initial activation at 95 °C for 10 min followed by 40 cycles of 95 °C for 15 s and 60 °C for 1 min. Ct values were retrieved by maintaining the same threshold among all the samples.

### 4.6. Data Analysis and Statistics

For the Oxidative Stress PCR Array (PAMM-065) and *p53* Signaling Pathway PCR Array, relative expression levels of genes were calculated using the comparative threshold cycle method (ΔΔCt) with normalization to the average expression levels of five housekeeping genes (SABiosciences Data Analysis Software). FLT samples were compared to GRD controls using Mann–Whitney U Test using *p* < 0.05 as the threshold of significance and sample size of n = 4 mice per group.

For *Nfe2l2* RT-PCR of heart samples, the change in expression levels were calculated using the ΔΔCt method with Hypoxanthine Phosphoribosyltransferase (*Hprt*) 1 as housekeeping gene. The log2 (linear) normalized expression from flight and ground control groups was used in the Mann–Whitney U test at *p* < 0.05, n = 4 mice per group.

### 4.7. Gene Network Predictions and Functional Analysis

To generate predictions on potential protein interactions, statistically significant differentially expressed genes from both PCR arrays were entered into the String DB protein-protein interaction database [75]. Experimental evidence, databases, and co-expression were used as sources to generate protein interaction networks. The interaction score was set to a threshold of “medium” (0.400).

Toppfun [76] was used to gain insight into whether the differentially expressed genes were linked to human disease phenotypes. Statistically significant differentially expressed genes were entered into the Toppfun database. The results were filtered using a Benjamini–Hochberg false discovery rate (FDR) threshold of *p* < 0.05 and a query to genome overlap of four or more genes.

## 5. Conclusions

In conclusion, our study revealed that spaceflight significantly altered cardiac expression of genes related to cell cycle/growth (notably *Cdkn1a*/*p21, Cdk1,* and *Myc*), inflammation (notably *Tnf*) and oxidative stress (notably *Nfe2l2*, *Nox1, Ptgs2*), which may contribute to cardiac dysfunction. These findings provide novel data suitable for developing testable hypotheses for further study. Identification of specific mechanisms and molecules responsible for excess ROS generation during spaceflight is of considerable interest, and may yield new therapeutic approaches.

## Figures and Tables

**Table 1:** Spaceflight altered the expression of genes involved in oxidative stress pathway. Expression levels are represented as fold regulation in spaceflight versus ground controls (FLT versus GRD). Shown are 14 differentially regulated genes out of a panel of 84 genes related to the oxidative stress response as measured by RT-PCR. Of these, 11 were upregulated while three were downregulated. The change in expression levels of these genes were found to be statistically significant by Mann–Whitney U test at *p* < 0.05, n = 4.

Gene Symbol	Gene Name	Fold Regulation
*Nox1*	NADPH oxidase 1	6.62
*Tpo*	Thyroid peroxidase	2.35
*Slc41a3*	Solute carrier family 41, member 3	1.97
*Txnip*	Thioredoxin interacting protein	1.94
*Prnp*	Prion protein	1.44
*Txnrd3*	Thioredoxin reductase 3	1.43
*Gsr*	Glutathione reductase	1.31
*Prdx6*	Peroxiredoxin 6	1.23
*Cat*	Catalase	1.20
*Xpa*	Xeroderma pigmentosum, complementation group A	1.18
*Sod1*	Superoxide dismutase 1, soluble	1.18
*Ncf2*	Neutrophil cytosolic factor 2	−1.49
*Fancc*	Fanconi anemia, complementation group C	−1.62
*Ptgs2*	Prostaglandin-endoperoxide synthase 2	−3.44

**Table 2:** Spaceflight altered the expression of genes involved in the *Trp53* signaling pathway. Expression levels are represented as fold regulation in spaceflight versus ground controls (FLT versus GRD). Shown are 23 differentially regulated genes out of a panel of 84 genes related to the *Trp53* signaling pathways as assessed by RT-PCR. Out of these, 13 genes were upregulated and 10 genes were downregulated. The change in expression levels of these genes were found to be statistically significant by Mann–Whitney U test at *p* < 0.05, n = 4.

Gene Symbol	Gene Name	Fold Regulation
*Cdkn1a*	Cyclin-dependent kinase inhibitor 1A (P21)	6.80
*Myc*	Myelocytomatosis oncogene	3.91
*Hif1a*	Hypoxia inducible factor 1, alpha subunit	1.58
*Pmaip1*	Phorbol-12-myristate-13-acetate-induced protein 1	1.54
*Prkca*	Protein kinase C, alpha	1.49
*Trp53bp2*	Transformation related protein 53 binding protein 2	1.41
*Apex1*	Apurinic/apyrimidinic endonuclease 1	1.28
*Ercc1*	Excision repair cross-complementing rodent repair deficiency, complementation group 1	1.27
*Foxo3*	Forkhead box O3	1.27
*Mlh1*	MutL homolog 1 (E. coli)	1.19
*Pten*	Phosphatase and tensin homolog	1.18
*Xrcc5*	X-ray repair complementing defective repair in Chinese hamster cells 5	1.13
*Atr*	Ataxia telangiectasia and rad3 related	1.10
*Cdk4*	Cyclin-dependent kinase 4	−1.16
*Apaf1*	Apoptotic peptidase activating factor 1	−1.41
*Tnfrsf10b*	Tumor necrosis factor receptor superfamily, member 10b	−1.92
*Chek2*	CHK2 checkpoint homolog (*S. pombe*)	−2.15
*Traf1*	Tnf receptor-associated factor 1	−2.25
*Jun*	Jun oncogene	−2.31
*Pttg1*	Pituitary tumor-transforming gene 1	−2.42
*Btg2*	B-cell translocation gene 2, anti-proliferative	−3.50
*Cdk1*	Cyclin-dependent kinase 1	−4.11
*Tnf*	Tumor necrosis factor	−9.86

## Data Availability

All data associated with this study are provided in the main figures and tables and in the supplementary information.

## Supplementary Materials

The following are available online at https://www.mdpi.com/article/10.3390/ijms22169088/s1.
